# HSP60, a protein downregulated by IGFBP7 in colorectal carcinoma

**DOI:** 10.1186/1756-9966-29-41

**Published:** 2010-04-30

**Authors:** Wenjing Ruan, Yinghong Wang, Yu Ma, Xiaoming Xing, Jie Lin, Jing Cui, Maode Lai

**Affiliations:** 1 Department of Pathology, School of Medicine, Zhejiang University, 388 Yuhangtang Road, Hangzhou 310058, Zhejiang Province, China; 2 Department of Respiratory Diseases, Sir Run Run Shaw Hospital, Zhejiang University, 3 East Qingchun Road, Hangzhou 310016, China

## Abstract

**Background:**

In our previous study, it was well defined that *IGFBP7 *was an important tumor suppressor gene in colorectal cancer (CRC). We aimed to uncover the downstream molecules responsible for *IGFBP7*'s behaviour in this study.

**Methods:**

Differentially expressed protein profiles between PcDNA3.1(*IGFBP7*)-transfected RKO cells and the empty vector transfected controls were generated by two-dimensional gel electrophoresis (2-DE) and mass spectrometry (MS) identification. The selected differentially expressed protein induced by IGFBP7 was confirmed by western blot and ELISA. The biological behaviour of the protein was explored by cell growth assay and colony formation assay.

**Results:**

Six unique proteins were found differentially expressed in PcDNA3.1(*IGFBP7*)-transfected RKO cells, including albumin (ALB), 60 kDa heat shock protein(HSP60), Actin cytoplasmic 1 or 2, pyruvate kinase muscle 2(PKM2), beta subunit of phenylalanyl-tRNA synthetase(FARSB) and hypothetical protein. The downregulation of HSP60 by IGFBP7 was confirmed by western blot and ELISA. Recombinant human HSP60 protein could increase the proliferation rate and the colony formation ability of PcDNA3.1(*IGFBP7*)-RKO cells.

**Conclusion:**

HSP60 was an important downstream molecule of IGFBP7. The downregulation of HSP60 induced by IGFBP7 may be, at least in part, responsible for IGFBP7's tumor suppressive biological behaviour in CRC.

## Introduction

Colorectal cancer (CRC) is the third most common malignancy in the world. Colorectal carcinogenesis has been conceptualized as a multi-step, multi-mechanism process, consisting of an initiation, promotion and progression phase, which developed via a progressive accumulation of genetic mutations. Understanding the neoplastic progression of CRC at the cellular and molecular levels can facilitate diagnosis and treatment of cancer.

Our lab has been devoted to research on the molecular mechanism of CRC for decades of years. In 1999, we separated the insulin-like growth factor binding protein 7 (*IGFBP7*) cDNA fragments from colonic adenocarcinoma and normal mucosa cDNA subtraction libraries by suppressive subtractive hybridization (SSH)[1]. *IGFBP7 *was cloned as a senescence-associated gene from human mammary epithelial cells[2], also named as insulin-like growth factor binding protein-related protein 1 (*IGFBP-rP1*)[3], meningioma associated cDNA 25 (*MAC25*)[2,4], tumor-derived adhesion factor(*TAF*)[5], and prostacylin-stimulating factor(*PSF*)[6]. After the separation of *IGFBP7*, we then devoted to elaborate the biological role of the protein in CRC. Our group presented evidence that reintroduction of *IGFBP7 *suppressed the proliferation, decreased the colony formation ability, and induced apoptosis in two colorectal carcinoma cell lines RKO and SW620[7]. IGFBP7 protein could induce G1 cell cycle arrest in RKO and CW2 cells. A senescence-like phenotype was induced by IGFBP7 in these colon cancer cells[8]. We also found that overexpression of IGFBP7 in CRC tissue correlated with favourable prognosis, the higher IGFBP7 expressed the longer will the patient survive[7,9]. All these findings strongly supported that IGFBP7 played a potential tumor suppressor role against colorectal carcinogenesis. In consistent with our findings, the tumor suppressor roles of IGFBP7 in cervical cancer[10], osteosarcoma[10,11], prostate cancer[12,13], and breast cancer[14] were discovered by other laboratories.

The important function of IGFBP7 protein in CRC has elicited the need to further investigate the underlying mechanism. Proteomics represents a powerful approach to analyze alterations in protein expression in complex biological system. This approach has been used successfully in our lab to identify differentially expressed proteins between tissue of colorectal carcinoma, colon adenoma, and the normal mucosa, which have potential clinical interest [15,16]. In this study, our main goal was to identify proteins associated with IGFBP7 expression using the proteomics-based approach and further clarify the protein's biological role. These findings will contribute to our understanding for the molecular mechanism responsible for IGFBP7's tumor suppressive function in CRC.

## Methods

### Reagents

Dulbecco's Modified Eagle's Medium(DMEM)was purchased from GIBCO Laboratories (Grand Island, NY, USA). Fetal bovine serum (FBS) was purchased from HyClone Laboratories (Logan, UT, USA). Polyfect transfection reagent was purchased from QIAGEN (Hilden, Germany). G418 was purchased from Merck (Darmstadt, Germany). Immobiline Dry-Strips (17 cm, pH 3-10 NL), immobilized pH gradient (IPG) buffer, Dry-Strip cover fluid, urea, thiourea, ammonium bicarbonate and sodium dodecyl sulfate/polyacrylamide gel electrophoresis (SDS-PAGE) standards were purchased from BioRad (Hercules, CA, USA). Dithiothreitol (DTT), trifluoroacetic acid (TFA), acrylamide, cellulose acetate nitrate (ACN), glycerol, glycine, iodoacetamide3-((3-cholamidopropyl)dimethylammonio)-1-propanesulfonic acid (CHAPS), bis-hydroxymethyl-oxazoline (Bis), tetramethylethylenediamine (TEMED), sodium dodecyl sulfate (SDS), tris-hydroxymethyl-aminomethane (Tris base), dimethylsulfoxide (DMSO), bovine serum albumin (BSA) and Coomassie brilliant blue (CBB R-250) were obtained from Sigma Chemical (St. Louis, MO, USA). Cell lysis buffer, Glyceraldehyde-3-phosphate dehydrogenase (GAPDH) antibody, 60 kDa heat shock protein (HSP60) antibody, and horseradish peroxidase-linked second antibody were purchased from cell signaling Technology (Danvers, MA, USA). Recombinant human HSP60 protein and HSP60 ELISA kit were purchased from StressGen Biotechnologies (Victoria, British Columbia, Canada).

### Cell culture and protein extraction

Human colorectal carcinoma RKO cell lines were derived from the American Type Culture Collection (ATCC), maintained in DMEM supplemented with 10% FBS in a 37°C/5% CO_2 _atmosphere. RKO cells were transfected with either PcDNA3.1(*IGFBP7*) or an empty plasmid vector PcDNA3.1. Stable PcDNA3.1(*IGFBP7*)-RKO transfectants and PcDNA3.1-RKO transfectants were established as previously described[7]. For the proteomics analysis, the two groups of cells were cultured in the same conditions, maintained at 80% confluence and in exponential growth phase, harvested at the same time. Cells were washed with phosphate buffered saline (PBS) 3 times, solubilized in cell lysis buffer on ice for 30 min, followed by centrifugation at 100,000 g for 60 min at 4°C. The protein concentration was determined according to the method of Bradford. Samples were stored at -80°C.

### Two-dimensional electrophoresis (2-DE)

Briefly, linear gradient 24-cm (pH 5-8) readystrip (Bio-Rad) was rehydrated overnight at 17°C with 300 μg of protein samples in 500 μl of rehydration buffer (7 M urea, 2 M thiourea, 4% CHAPS, 65 mM DTT, and 0.2% Bio-Lyte). Isoelectric focusing (IEF) was performed by using PROTEAN IEF Cell (Bio-Rad). After IEF, the IPG strip was immediately equilibrated for 15 mins in equilibration buffer I (6 M urea, 2% SDS, 0.375 M Tris-HCl pH 8.8, 20% glycerol, and 2% DTT) and then for 15 mins in equilibration buffer II (6 M urea, 2% SDS, 0.375 M Tris-HCl pH 8.8, 20% glycerol, and 2.5% iodoaceta-mide). SDS-PAGE was carried out on 12% SDS-polyacrylamide gels (25 cm × 20.5 cm × 1.0 mm) by using the PROTEAN Plus Dodeca Cell (Bio-Rad) at a constant voltage of 200 V at 20°C. After electrophoresis, the gels were stained by using the Silver Stain Plus Kit (Bio-Rad). The above processes were performed in triplicate for each sample.

### Image Analysis

The silver-stained 2-DE gels were scanned on a GS-800 Calibrated Imaging Densitometer (Bio-Rad) at a resolution of 300 dots per inch (dpi). Spot detection, quantification, and the analyses of 2-D protein patterns were done with the PDQuest software (version 7.1, BioRad). Then the report of quantitative differences between two gel images was generated. The gray values of the differentially expressed protein candidates were statistically analyzed by the nonparametric Wilcoxon test. Protein spots that showed more than 3-fold differential expression reproducible in the three gels were taken as differentially expressed candidates and selected.

### Spot Cutting and In-Gel Digestion

Differentially expressed protein spots identified as described in the preceding text were excised from gels by Proteomeworks Spot Cutter (Bio-Rad), destained for 20 mins in 30 mM potassium ferricyanide/100 mM sodium thiosulfate (1:1 [v/v]), and washed in Milli-Q water until the gels shrank and were bleached. The gel pieces were incubated in 0.2 M NH_4_HCO_3 _for 20 mins and dried by lyophilization. To each gel piece, 20 μl of 20 μg/ml trypsin (proteomics grade, Sigma, St. Louis, MO) was added and incubated at 37°C overnight. The peptides were extracted three times with 50% ACN and 0.1% TFA and dried in a vacuum centrifuge.

### Matrix-assisted laser desorption ionization/time-of-flight mass spectroscopy (MALDI-TOF-MS) analysis and database search

Samples were analyzed by a Voyager-DE STR MALDI-TOF mass spectrometer (Applied Biosystems, Foster City, CA) with delayed extraction in which α-cyano-4-hydroxycinnamic acid was exploited as the matrix (in 50% ACN in 0.05% MS). The total 2 μl solution was applied onto a target disk and allowed to air dry. Mass-to-charge ratios were measured in a reflector/delayed extraction mode with an accelerating voltage of 20 kV, a grid voltage of 63%-65%, positive polarity, and a delay time of 200 nanoseconds. Laser shots at 300 per spectrum were used to acquire the spectra with a range from 800 to 4000 Daltons. Trypsin autolysis products were used for internal mass calibration. Database searching was performed by using Mascot software http://www.matrixscience.com. The search parameters were the nrNCBI database, human, 10-150 kDa, trypsin (1 missed enzymatic cleavage), and 100-ppm mass tolerance. The best match was the one with the highest score, and a significant match was typically a score of the order of 70 (P < 0.05) [16,17].

### Western blot

Cell lysates (50 μg) were loaded onto 12% SDS-polyacrylamide gels, transferred onto nitrocellulose membranes, and subjected to western blot analysis[7]. The transferred membranes were incubated overnight at 4°C with rabbit polyclonal antibodies against HSP60 at 1:1000 dilutions. The membranes then were washed three times in Tris Buffered Saline with Tween (TBST). Bands were detected using a horseradish peroxidase-linked second antibody and enhanced chemiluminescence reagents, according to the manufacturer's protocol.

### Enzyme-linked immunosorbent assay (ELISA)

Equivalent numbers 1 × 10^6 ^of PcDNA3.1(*IGFBP7*)-RKO transfectants and PcDNA3.1-RKO transfectants (control) were plated in 6-well plates. After attachment, the media were then changed to 1.5 ml of serum-free media and allowed to incubate on the cells for additional 24 h. The cell supernatants were then collected, centrifuged to discard cellular debris, and analyzed using HSP60 ELISA kit as recommended by the manufacturer.

### Cell proliferation assay

Cell proliferation was measured using the cell counting kit-8 (CCK-8, Dojindo Laboratories, Japan). In brief, PcDNA3.1(*IGFBP7*)-RKO cells were plated in sextuple in 96-well microtitre plates at 3 × 10^3^/well, cultured with medium with or without recombinant HSP60 protein(1 μg/ml). Ten μl of CCK8 was added to each well at the time of harvest (12 h, 24 h, 36 h, 48 h, 60 h, 72 h). Two hours after adding CCK8, cellular viability was determined by measuring the absorbance of the converted dye at 450 nm.

### Anchorage-independent growth assay

PcDNA3.1(*IGFBP7*)-RKO cells (500/well) were seeded into 0.3% Bacto-agar (Sigma, St Louis, MO, USA) over a 0.6% agar bottom layer in triplicate in 6-well plates, with or without 1 μg/ml HSP60. Plates were incubated in a 37°C/5% CO_2_, humid atmosphere for 3 weeks. Colonies were counted using a dissecting microscope. The wells were then analyzed for colony number and size. Colonies >100 μm in diameter were counted under a dissecting microscope. Three independent experiments were conducted.

### Statistical Analysis

All experiments were done in triplicate. Differences between control and treated cells were assessed using one-way ANOVA and a significance level of P < 0.05 was required.

## Results

### Comparative proteomics analysis

**Figure 1:**
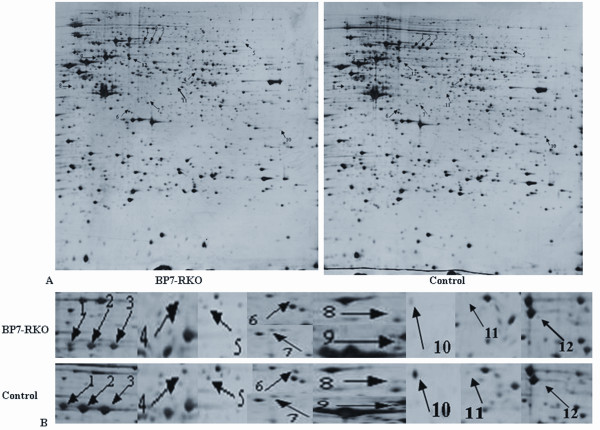
**2D electrophoresis profiles of PcDNA3.1(*IGFBP7*)-RKO-transfectants and PcDNA3.1-RKO transfectants**. A. 2D electrophoresis profiles of silver staining proteins of PcDNA3.1(*IGFBP7*)-RKO transfectants (BP7-RKO) and PcDNA3.1-RKO transfectants (control). 0.75 milligrams of protein were loaded onto linear IPG strips (pH 5-8) and isoelectric focusing was performed at 35 kV-h. The second dimensional run was performed on 12.5% Tris-glycine-PAGE gels and the gels were stained with silver for image analysis. Protein spot discrepancies were arrowed and marked with number. B. Close-up image of differential expression of protein spots.

The silver-stained 2D-PAGE profile of the PcDNA3.1(*IGFBP7*)-RKO transfectants and the PcDNA3.1-RKO -transfectants revealed approximate 1100 staining spots (1171 ± 109 vs 1120 ± 80), respectively. Using a 3-fold criterion for selecting, 12 protein spots were visually detected as significantly differentially expressed between the two groups. The representative images, emphasizing the location of the 12 protein spots on the gel were shown in Figure 1. Interestingly, of the 12 spots, only one spot was upregulated (spot 12) and the other 11 spots were downregulated in the cell lysates of PcDNA3.1(*IGFBP7*)-RKO transfectants.

### MS based identification

The above 12 differentially expressed protein spots were selected and submitted to MS based identification. As a result, 10 spots were identified by MALDI-TOF MS, representing 6 unique proteins, including albumin (ALB), HSP60, Actin cytoplasmic 1 or 2, pyruvate kinase muscle 2(PKM2), beta subunit of phenylalanyl-tRNA synthetase(FARSB) and hypothetical protein (Table 1). Two protein spots (spot 11 and spot 12) could not be identified, possibly due to the lower amount of protein as revealed by a retrospective analysis of the spot volumes. Of the 6 proteins identified above, all were found decreased in PcDNA3.1(*IGFBP7*)-RKO transfectants.

**Table 1:** Characteristics of proteins identified from PcDNA3.1(IGFBP7)-transfected RKO cells and controls

Spot	Protein description	Sequence coverage(%)*	Swissprot ID	Theoretical Mr/Pi**
1	Serum albumin	5.74%	P02768	69367/6.42
2	Serum albumin	7.97%	P02768	69367/6.42
3	Serum albumin	6.86%	P02768	69367/6.42
4	pyruvate kinase, muscle	22.45%	Q9UK31	6002/7.58
5	Phenylalanyl-tRNA synthetase beta chain	12.56%	Q9NSD9	66130/6.39
6	Actin, cytoplasmic 1 or 2	33.33%	P63261	41793/5.31
7	Actin, cytoplasmic 1 or 2	23.20%	P63261	41793/5.31
8	60 kDa heat shock protein, mitochondrial precursor	2.96%	P10809	61055/5.7
9	60 kDa heat shock protein, mitochondrial precursor	28.52%	P10809	61055/5.7
10	Hypothetical protein	21.49%	P04406	36053.05/8.57

* Sequence coverage was calculated by amino acid count **Calculated from the database entry without any processing

### Confirmatory studies of downregulation of HSP60 by Western blot and ELISA

**Figure 2:**
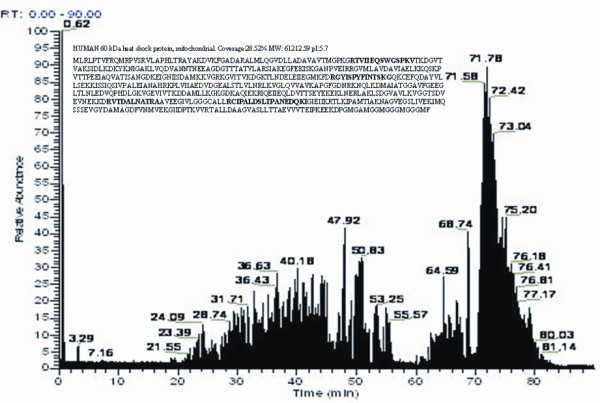
**LC-MS spectrum obtained for spot 9**. Peptide fragments were analyzed by LC-tandem MS and MALDI-TOF analysis of peak m/z was performed. Major monoisotopic peaks of trypsin-digested peptides, detected by MALDI-TOF MS, are indicated on the spectrum. The sequence of HSP60 protein was represented by single-letter code for amino acids on the top left corner of the image, where peptide matches between the sample and the HSP60 sequence are shown bold.

**Figure 3:**
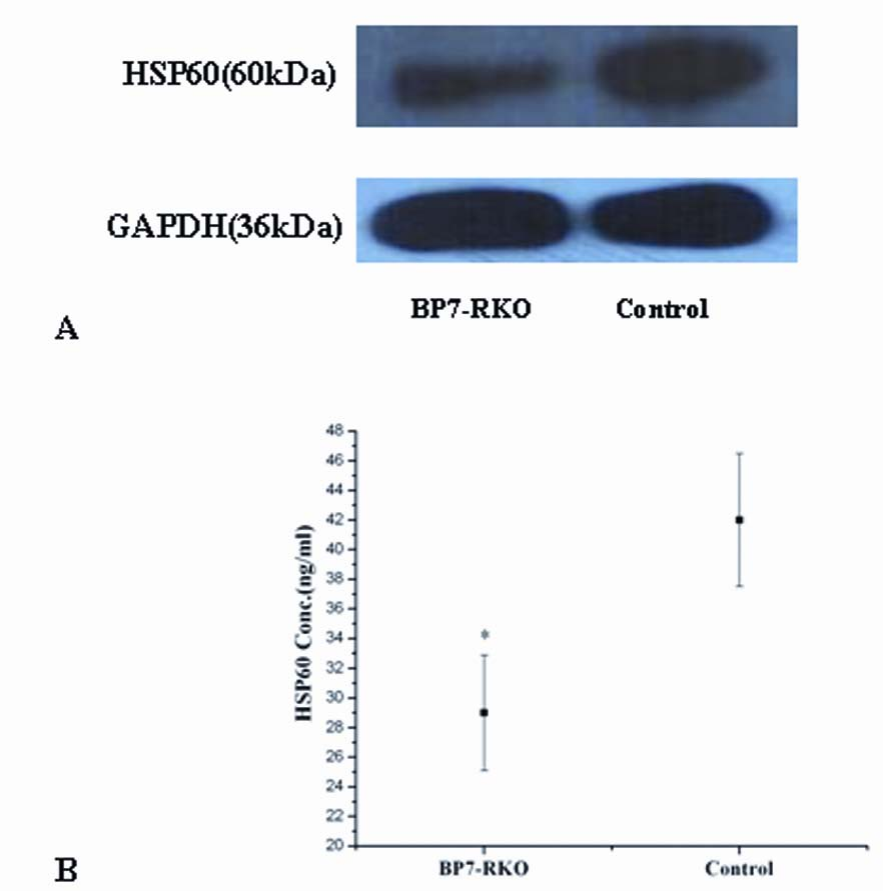
**Downregulation of HSP60 protein expression in PcDNA3.1(*IGFBP7*)-RKO transfectants**. A: Whole cell lysates of the stable PcDNA3.1(*IGFBP7*)-RKO transfectants and PcDNA3.1-RKO transfectants were prepared, and equal amounts of protein (50 μg/lane) were loaded. HSP60 expression was assessed using a rabbit anti-HSP60 antibody. GAPDH is used as an internal loading control. Shown is representive of experiments performed on at least three different isolations. The amount of HSP60 protein expression in PcDNA3.1(*IGFBP7*)-RKO transfectants was lower than that of the control group. B: HSP60 concentration in cell supernatants was measured using the HSP60 ELISA kit according to the manufactures instructions. Experiments were performed in triplicates. Results represent the mean HSP60 concentration (ng/ml) ± SD,. *, p < 0:05 vs. control.

In particular, we focused on the protein spot 8 and 9. We identified these two spots as the HSP60, with a molecular weight of 61.055 kDa, and a pI value of 5.70. The spectrum figure of HSP60 was presented in Figure 2. Western blot results using the cell lysates samples confirmed the findings that the expression of HSP60 was significantly lower in the cell lysates of PcDNA3.1(*IGFBP7*)-RKO transfectants. A representative image was presented in Figure 3A. The secretion of HSP60 was also compared between the supernatants from PcDNA3.1(*IGFBP7*)-RKO transfectants and controls using ELISA. Secretion of HSP60 was also found to be downregulated by IGFBP7 (Figure 3B).

### Recombinant HSP60 reversed the proliferation inhibition induced by IGFBP7

**Figure 4:**
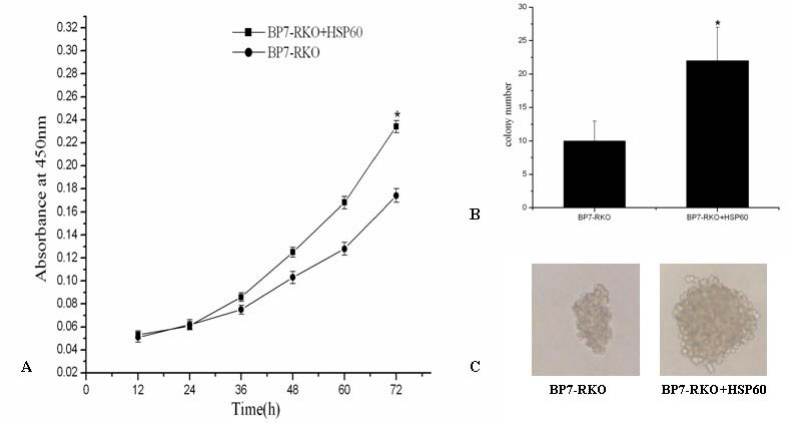
**HSP60 protein decreased the proliferation rate and colony formation ability of PcDNA3.1(*IGFBP7*)-RKO cells**. A: PcDNA3.1(*IGFBP7*)-RKO cells were plated in sextuple in 96-well microtitre plates at 3 × 10^3^/well, cultured with medium with or without recombinant HSP60(1 μg/ml). Ten μl of CCK8 was added to each well at the indicated time (12 h, 24 h, 36 h, 48 h, 60 h, 72 h). Cellular viability was determined by measuring the absorbance of the converted dye at 450 nm two hours after adding CCK8. Values shown are mean ± SD for sextuple cultures from one experiment, representive of three independent experiments conducted. B: PcDNA3.1(*IGFBP7*)-RKO cells (500/well) were seeded into 0.3% Bacto-agar over a 0.6% agar bottom layer in triplicate in 6-well plates, with or without 1 μg/ml HSP60. After 3 weeks of incubation, colony number (>100 μm)were analyzed. Values are mean ± S.D for data from three independent experiments. C: Colony size was also analyzed under microscopy. Representive size of the colony was photographed under high power microscopy (×100).

To clarify the biological effect of HSP60 downregulation induced by IGFBP7 in RKO cells, we studied the function of recombinant HSP60 on the proliferation of PcDNA3.1(*IGFBP7*)-RKO cells. We found that addition of HSP60 protein could promote the cell proliferation rate of PcDNA3.1(*IGFBP7*)-RKO cells(Figure 4A). HSP60 could also increase the colony formation ability and the colony size of the cells (Figure 4B, 4C).

## Discussion

Here we describe a proteomics study of two human colon cancer cell lines differing in the expression of IGFBP7, which is an important tumor suppressor gene well defined by our previous studies[7]. To our knowledge, this is the first proteomic study on the alterations of IGFBP7 protein expression profiles in colon cancer cells. We were successful in identifying six IGFBP7-associated downstream target proteins, including ALB, HSP60, Actin cytoplasmic 1 or 2, PKM2, FARSB and hypothetical protein. These differentially expressed proteins represent candidate proteins that may be directly or indirectly regulated by IGFBP7.

The comparation between the current findings at the translation level and our previous studies identifying the IGFBP7-induced genes at the transcriptional level detected by Affymetrix chip platform(unpublished data) resulted in some interesting points in agreement. The proteomics finding indicated that actin was influenced by IGFBP7. While the cDNA array studies also indicated that the actin binding proteins were greatly influenced by IGFBP7. These findings at both the transcriptional and the translational level suggested that *IGFBP7 *may possibly be an actin-binding associated gene, which need our further study to provide the direct evidence.

However, there is little overlap of identified genes between our mRNA and protein data, consistent with the data reviewed by Sagynaliev and the colleagues that among various gene expression studies only about 25% of differentially expressed proteins were reflected by concomitant changes at the mRNA level in CRC [18]. This may be due to two reasons. First, the lower dynamic range of the 2D PAGE protocol allows less abundant proteins to escape detection [19]. With only around 1100 protein spots visible, this approach allows the analysis of only a fraction of the total number of proteins expressed in the cell. Second, from the transcriptional profiles, we found that IGFBP7 could influence the expression levels of many secretary genes. However, many of them could not be detected by the current proteomics approach in the cell lysates samples. Secretome studies performed in the supermedium of the cells will probably enlarge our finding [20].

Among the differentially expressed proteins induced by IGFBP7, HSP60 attracted our attention. Heat shock proteins (HSPs) are a family of highly conserved proteins induced by various kinds of stress, mostly localized in the mitochondrial matrix, including HSP60, HSP90, HSP70 and small HSPs. They are with the principal function as molecular chaperones results in the maintenance of stability and delivery of other peptide [21]. Recently, HSPs are implicated in several important cellular processes, including DNA replication, gene expression regulation, signal transduction, differentiation, apoptosis, or immortalization[22]. Our data obtained from western blot using the cell lysates confirmed the proteomics finding that HSP60 was downregulated in PcDNA3.1(*IGFBP7*)-RKO transfectants. Similar with the secretary character of IGFBP7, in addition to the cytosolic locations, HSP60 also could be detected in the extracellular space and in circulation[23,24]. Thus, we also analysed the secretion of HSP60 in the supernatants of the cells using ELISA. Consistent with the expression level in the cell lysates, it was found that the IGFBP7 could also decrease the secretion of HSP60 in RKO cells.

The role of HSP60 played in cancer has been investigated by numerous studies. Strong patterns of increased HSP60 immunostaining from normal tissues, through cervical intraepithelial neoplasia grade (CIN)1, to CIN3 was found, in a manner similar to cyclin-dependent kinase inhibitor 2A (CDKN 2A), a biomarker of oncogenic human papillomaviruses (HPV) infections and CIN3[25]. In breast cancer, HSP60 expression gradually increased from normal through ductal carcinoma in situ (DCIS) to invasive tissues [26]. HSP60 expression was significantly increased in both early and advanced prostate cancer compared with nonneoplastic prostatic epithelium[27]. The upregulation of HSP60 in leukemia was associated with major adverse prognostic factors in acute myeloid leukemia [28]. The upregulation of HSP60 in these cancerous tissue may be functionally correlated to tumor initiation and progression. *In viro*, the survival-promoting effects of HSP60 *in vitro *has also been reported. HSP60 was detected in exosomes purified from culture media of H292, A549 and K562 tumor cell lines, while not in the non tumor 16HBE cells, suggesting the spontaneous release of this molecule usually occurs in tumor cells[29]. HSP60 could mediate the nuclear factor kB (NF-Kb) dependent survival signaling in the cells[30]. Acute ablation of HSP60 in tumor cells results in loss of the mitochondrial pool of survivin and activation of p53-dependent apoptosis [31]. Cytosolic HSP60 is associated with procaspase-3 in the apoptosis systems, including HCT116 cells stimulated with Fas cross-linking antibody, LNCaP cell treated with doxorubicin (Dox), or PC3 cells treated with staurosporine (STS). Knockdown of HSP60 enhances caspase activation and cell death, suggesting the antiapoptotic role of HSP60/procaspase-3[32]. Upon oxidative stress, the antiapoptotic Hsp60/procaspase-3 complex persists in mucoepidermoid carcinoma cells[33].

However, the role of HSP60 is context based. There is some data in contrast. Downregulation of HSP60 was found in prostate cancer[34]and lung cancer[35]. Positive HSP60 expression in esophageal squamous cell carcinoma[36], ovarian cancer [37] and bladder cancer[38] correlated with good prognosis for the patients. Mechanistic studies in different cell models indicated that association of HSP60 with procaspase-3 promotes caspase-3 maturation and activation, suggesting a pro-apoptotic role[32,39,40].

In the past decades, regarding HSP60's roles in CRC, most of the data come from expression observations. As shown by immunohistochemistry, western blot[41-43] and by cDNA microarray analysis[44,45], it was found that HSP60 was overexpressed in CRC tissue. The levels of HSP60 correlated with tumor grade and stage and with occurrence of lymph node metastases[44]. While the data on the exact biological function of HSP60 in CRC cells is still lack. In this study, to clarify the biological role of the down-regulation of HSP60 induced by IGFBP7, we also explored the function of HSP60 protein in PcDNA3.1(*IGFBP7*)RKO cells. We found that addition of recombinant HSP60 could increase the proliferation rate and increase the colony formation ability of PcDNA3.1(*IGFBP7*)-RKO cells. The studies provide the evidence that 1. HSP60 protein may be a key molecule enrolled in CRC initiation and progression. 2. Downregulation of HSP60 may participate in, at least in part, the growth inhibiting role of IGFBP7 on colon cancer cells. However, the exact underlying molecular mechanism is still unclear. Both IGFBP7 and HSP60 could influence the extracellular signal pathways. Wajapeyee et al. reported that secretion of IGFBP7 acted through autocrine/paracrine pathways to inhibit mitogen-activated protein kinase (MAPK)- extracellular signal -regulated kinase (ERK) signaling [46]. Zhang et al. reported that HSP60 protected epithelial cells from stress-induced death through activation of ERK and inhibition of caspase 3 [47]. Whether HSP60 is complexed with pro-caspase 3 and influenced the caspase 3 and ERK signaling in colon cancer cells will remain an active subject of our ongoing research.

## Conclusion

We have identified six candidate proteins whose expression were downregulated by reintroduction of IGFBP7 in the colon cancer RKO cells using a proteomics approach. These results contributed to our better understanding of the potential underlying molecular mechanism for IGFBP7's tumor suppressive role in CRC. Downregulation of HSP60 may be responsible for, at least in part, the proliferation inhibiting role of IGFBP7 in colorectal cancer cells. Further studies are warranted to elaborate the exact biological role and the molecular mechanism for HSP60 in colorectal carcinogenesis.

## Competing interests

The authors declare that they have no competing interests.

## Authors' contributions

RWJ carried out the design of the study, performed the cell growth assay, soft agar colony formation assay, western blot and ELISA assay, drafted the manuscript and participated in the proteomics study. WYH performed the two-dimensional gel electrophoresis, participated in mass spectrometry identification assay. MY participated in the cell culture, protein extraction and two-dimensional gel electrophoresis assay. XXM participated in the two-dimensional gel electrophoresis study. LJ participated in the mass spectrometry identification assay. CJ participated in the cell culture and ELISA assay. LMD participated in the design of the study, carried out the statistical analysis and helped drafting the manuscript. All authors read and approved the final manuscript.
